# Outcomes of Pulmonary Arterial Hypertension in Pregnancy: A Tertiary Care Retrospective Study

**DOI:** 10.7759/cureus.93463

**Published:** 2025-09-29

**Authors:** Seema Chopra, Amanjot Kaur, Rakhi Rai, Pooja Sikka, Neelam Aggarwal, Vanita Suri, Rajesh Vijayvergiya

**Affiliations:** 1 Obstetrics and Gynaecology, Postgraduate Institute of Medical Education and Research, Chandigarh, Chandigarh, IND; 2 Cardiology, Postgraduate Institute of Medical Education and Research, Chandigarh, Chandigarh, IND

**Keywords:** heart disease, maternal mortality, pregnancy, prostacyclin, pulmonary arterial hypertension

## Abstract

Background and aims: Pulmonary arterial hypertension (PAH) is a rare disease affecting mainly women of childbearing age. It is associated with high maternal mortality. Hence, obstetricians and cardiologists recommend against pregnancy, thereby stressing the need for effective contraception such as an intrauterine contraceptive device (IUD), and in case pregnancy occurs, early termination of pregnancy should be considered. The present study aims to retrospectively analyse 12 cases presenting with PAH in pregnancy and their maternal and fetal outcomes.

Methods: Retrospective analysis of 12 cases of PAH in pregnancy who reported to the emergency or in the cardio-obstetric clinic of Postgraduate Institution of Medical Education and Research (PGIMER), Chandigarh, India, in the last 24 years was conducted in terms of obstetric, cardiology, and neonatal parameters. PAH was diagnosed on the basis of high systolic pulmonary artery pressure (sPAP) > 20 mmHg on echocardiography. Secondary causes of pulmonary hypertension were ruled out in all patients whenever possible, depending on maternal condition.

Results: Out of the total 12 patients, 10 (83.33%) presented in the antepartum period, and two (16.66%) in the postpartum period. Moreover, 50% were New York Heart Association (NYHA) class 3-4 at admission. There was increased PAP, ranging from 42 to 140 mmHg. As regards obstetrical outcome in 10 patients who delivered in our institution, one (10%) delivered vaginally, five (50%) had a cesarean section, one (10%) underwent medical termination of pregnancy (MTP), one (10%) patient was lost to follow-up, and two (20%) died undelivered. Maternal mortality was 36.36% (4/11), which included two antenatal and two delivered patients. Out of seven patients who delivered, five (71.42%) had fetal growth retardation, and one (14.28%) had stillbirth.

Conclusion: PAH has high maternal mortality despite modern treatment modalities. Patients presenting with higher NYHA class had poorer outcomes and a higher incidence of fetal growth retardation. Hence, MTP should be offered to patients with PAH who present in early pregnancy. A multidisciplinary (cardiology, anaesthesiology, neonatology, cardiac anaesthetist, along with an obstetrician experienced in managing high-risk pregnancy) team approach should be adopted while managing such patients. However, considering a very high risk in continuing the pregnancy, an ethical dilemma for continuing the pregnancy in women desirous of the same remains.

## Introduction

Pulmonary arterial hypertension (PAH) is a very rare, progressive, and incurable disease of the pulmonary vessels, causing high pressure in small arteries and arterioles of the lung, leading to right heart failure. When associated with pregnancy, it carries a very poor prognosis with a mortality rate ranging from 30% to 50% [1]. In 1973, the reported survival was less than three years. Several treatment modalities have now come up, which have doubled the survival time, and a larger number of women are becoming pregnant with the condition. Still, pregnancy complicated by PAH increases risks to the mother, which may have fatal outcomes [2]. Therefore, obstetricians and cardiologists recommend against pregnancy in view of high maternal-fatal mortality associated with PAH, emphasising the need for effective contraception, and if pregnancy occurs, early termination is important [3].

Labor and delivery place a lot of strain on the heart. Death often occurs during delivery or the early postpartum period because of right ventricular failure secondary to increased pulmonary vascular resistance coupled with physiologic changes of labor. In view of the very low incidence (two cases per million in the general population) [1], there is very little literature to guide management for patients who present with PAH in pregnancy. Targeted therapy for PAH involves the use of phosphodiesterase type 5 inhibitors (e.g., sildenafil, tadalafil), guanylate cyclase stimulants (e.g., riociguat), prostacyclins (e.g., epoprostenol, treprostinil, iloprost, selexipag), and endothelin receptor antagonists (e.g., bosentan, macitentan, ambrisentan). Treatment mandates a multidisciplinary team approach involving the obstetrician, cardiologist, and anaesthetist. We hereby present our experience of 12 patients of pregnancy with PAH presenting to our hospital in the last 25 years (2000-2024), which may be the largest cohort of patients from a developing country. In this study, we have attempted to analyse maternal and fetal outcomes and their key predictors, assess the efficacy of targeted therapies, and analyse factors influencing the outcomes in pregnant patients presenting with PAH.

## Materials and methods

The data were retrospectively collected from the records of the Department of Obstetrics and Gynaecology of the Institution. The study included 12 cases of PAH.

Inclusion criteria: Patients with high systolic pulmonary artery pressure (sPAP) confirmed on echocardiography with no secondary cause for PAH and who gave consent for participation in the study. The consent was taken retrospectively from the patients/ attendants.

Exclusion criteria: The patients who had secondary causes for PAH and/or those who did not consent to the study were excluded.

A standardised checklist was used for recording the data. The baseline characteristics, such as age, parity, period of gestation at presentation and delivery, New York Heart Association (NYHA) functional class(on clinical assessment), oxygen saturation (on atrial blood gas analysis), comorbidities, and medications, were recorded. Secondary causes were excluded after various relevant investigations, which included pulmonary function tests, HIV serology, autoimmune evaluation, liver function tests, electrocardiography, arterial blood gases, chest X-ray (followed by a high-resolution CT scan if interstitial lung disease is suspected), and a ventilation-perfusion scan to exclude chronic thromboembolic cause. Patient management was done in collaboration with the cardiology department. Arterial blood gas monitoring was done during antenatal visits and managed with oxygen by mask when oxygen saturation was low. Anticoagulants were started when the hematocrit was more than 45%. The treatment modalities used, such as vasodilators, sildenafil, oxygen inhalation or anticoagulants, need for intubation, and invasive cardiac monitoring, such as central venous line (CVP) or arterial line insertion, were recorded. Maternal-fetal outcomes in terms of mode and timing of delivery, labor analgesia, anesthesia used, maternal and perinatal morbidity and mortality, Apgar scores, and neonatal intensive care (NICU) stay were noted in all patients. Approval was taken from the institutional review board for conducting this retrospective study (IEC-INT/2025/Study-2927).

Statistical analysis of this retrospective study was limited to descriptive statistics, including frequencies and percentages, in view of the small sample size (12 patients).

## Results

Twelve patients with PAH in pregnancy were identified by analysing the hospital records and were included in the study. Ten (83.33%) patients presented in the antepartum period (three in the first trimester, one in the midtrimester, and six in the third trimester), and two (16.66%) in the postpartum period (day 12 and day 3 postpartum). Six (50%) patients were in right heart failure (NYHA class 3-4).

Table 1 shows the demographic profile and obstetric details of the patients. Management of the patients and cardiac evaluation and results are tabulated in Table 2. In cases 2 and 7, CT pulmonary angiography (CTPA) was done prior to pregnancy, and in cases 1, 3, 4, 8, and 11, CTPA was done after presentation. Eight (66.66%) patients belonged to the age group of 21-30 years. Six (50%) patients were primigravidas. Oxygen saturation varied from 68% to 99%. Echocardiography revealed gross right ventricular dilatation and increased pulmonary artery pressure ranging from 42 to 140 mmHg. Amongst the 10 patients who presented in the antepartum period, one (10%) delivered vaginally (case 5), five (50%) had cesarean section (cases 2, 9-12), one (10%) underwent MTP (case 7), one (10%) patient had unknown outcome, one was lost to follow up (case 3), and two (20%) died undelivered (cases 6 and 8). One (10%) patient (case 9) had a mortality after cesarean section. Three (30%) patients delivered at term, and five (50%) had preterm delivery at 34 weeks (cases 1 and 5), 32 weeks (case 9), and 36 weeks (cases 10 and 11). Out of seven patients who delivered, five (71.42%) had intrauterine growth retardation. Four (36.66%) out of 11 patients died (two postpartum and two undelivered), and one (14.28%) patient had stillbirth.

**Table 1:** Evaluation and management of the patients O2 - oxygen, sPAP- systolic pulmonary artery pressure, RV dilatation - right ventricular dilatation, CTPA - CT pulmonary angiography, NYHA - New York Heart Association

S. No.	NYHA class at presentation	Cyanosis	O2 saturation	Chest X-ray	sPAP on ECHO	RV dilatation	CTPA (e/o thrombosis)	Medical Management
Case 1	3	+	68%	Left lower lobe collapse, dilated pulmonary artery	90	Gross	Absent	Anticoagulation, O2
Case 2	1	-	94%	-	103	Gross	Absent	Anticoagulation
Case 3	2	+	88%	-	92	Gross	Absent	Anticoagulation, O2
Case 4	3		96%	Prominent pulmonary conus, peripheral pruning, cardiomegaly	89	Gross	Absent	Anticoagulation, O2, antibiotics
Case 5	2	+	99%	Pulmonary trunk dilatation	140	Gross	-	Anticoagulation, O2
Case 6	4	+	94%	-	99	Gross	Not done	Anticoagulation, O2
Case 7	1	-	98%	Pulmonary trunk dilatation	100	Mild	Absent	Anticoagulation, sildenafil
Case 8	4	+	90%	Pulmonary trunk dilatation	108	Gross	Absent	Anticoagulation, O2
Case 9	3	+	74%	Pulmonary trunk dilatation	121	Gross	Not done	Anticoagulation, sildenafil, milrinone, tadalafil, selexipag, ambrisentan
Case 10	3	-	96%	Pulmonary trunk dilatation	72	Gross	Not done	Sildenafil, ambisertan, tadalog
Case 11	2	-	96%	-	127	Gross	Absent	Sildenafil, selexipag
Case 12	2	-	96%	-	42	Mild	Not done	Sildenafil

**Table 2:** Demographic profile and obstetric details of the patients * APGAR score - appearance, pulse, grimace, activity, respiration score of newborn at birth

S. No.	Age (years)	Parity	Time of presentation	Gestation at delivery	Mode of delivery	Type of anesthesia	Birth weight (Kg)	APGAR^* ^score	Outcome
Case 1	20	P1A1	D12 postpartum	34 weeks	Vaginal delivery	-	1.2	0,0	Died at 14 hours of admission
Case 2	31	Primigravida	Antepartum 7+3 weeks	38 weeks	Cesarean section	General anaesthesia	2.0	8,9	Discharged in 7 days
Case 3	25	Primigravida	Antepartum 36+1 weeks	-	-	-	-	Unavailable	Lost to follow up, pregnancy outcome not known
Case 4	36	P3L3	D2 Postpartum	Term	Vaginal delivery	-	2.5	Unavailable	Improved on treatment, discharged satisfactorily
Case 5	22	Primigravida	Antepartum 29 weeks	34 weeks (had preterm labor)	Vaginal delivery	-	0.823	8,9	Discharged uneventfully
Case 6	23	G3P1L1A1	18 weeks	undelivered	Undelivered	-	Undelivered	Inapplicable	Died after few hours of admission
Case 7	27	Primigravida	7 weeks	MTP @ 8wks	Suction & evacuation	General anaesthesia	MTP	Inapplicable	Discharged satisfactorily after Suction & Evacuation
Case 8	32	G5P2L2A2	35 weeks	Undelivered	Undelivered	-	Undelivered	Inapplicable	Died after a few hours of admission
Case 9	32	G4P1020	28 weeks	30 weeks 6 days	LSCS	General anaesthesia	980 g	4,7,9	Had cardiac arrest on the 4th postoperative day, and could not be revived
Case 10	30	G2P1L1	5 weeks	36 weeks	LSCS	GA	2.804 kg	8,9	Discharged on the 10^th^ day after surgery
Case 11	24	Primigravida	36 weeks	36 weeks	LSCS	SA	1.020 kg	6,8	Discharged on the 6^th^ day after surgery
Case 12	29	Primigravida	Before pregnancy	37 weeks	LSCS	SA	2.8 kg	8,9	Discharged on the 7^th^ day after surgery

## Discussion

PAH is defined as increased PAP (mean pressure: >20 mmHg) in the absence of any demonstrable cause [1]. All our patients had increased PAP of more than 20 mmHg, ranging from 42 to 140 mmHg. The pathophysiology of PAH involves pulmonary arteriopathy, vascular remodeling, vasoconstriction, and thrombosis in the pulmonary vasculature. Histologically, there is medial hypertrophy, intimal fibrosis, fibrinoid necrosis, arteritis, and plexiform lesions in the pulmonary vasculature [4]. This high pressure leads to vasoconstriction of blood vessels in the lungs, ultimately causing the fibrosis of affected blood vessels. The increased pulmonary vascular pressure within the lungs impairs the blood flow and leads to right ventricular hypertrophy and decreased ability of the heart to pump blood through the lungs, causing right heart failure. Hence, the left side of the heart receives not only less but also poorly oxygenated blood, supplying insufficient oxygen to the rest of the body [2].

PAH is a rare disease affecting mainly women of childbearing age [4]. Vasoconstriction due to the reduction of nitric oxide and prostacyclin, along with an increase in endothelin and thromboxane in the vascular endothelium and smooth muscles, is important in the pathogenesis. It may present for the first time during pregnancy. Additionally, 70% of our patients were diagnosed in pregnancy or in the postpartum period, and 5/11 (45.45%) were already diagnosed with the condition prior to pregnancy. Sudden onset of dyspnea, syncope, or chest pain during pregnancy/postpartum period should be immediately investigated. Sleep apnea, cardiomyopathy, restrictive and interstitial lung disease, asthma, chronic obstructive pulmonary disease, arteriovenous malformations, atrial myxoma, amniotic fluid embolism, emphysema, atrial septal defect, mitral regurgitation and stenosis, and systemic lupus erythematosus are differential diagnoses [2].

Investigations to exclude other differential diagnoses include pulmonary function tests, HIV serology, autoimmune evaluation, liver function tests, electrocardiography, arterial blood gases, chest X-ray (followed by a high-resolution CT scan if interstitial lung disease is suspected), and a ventilation-perfusion scan to exclude chronic thromboembolic causes. In our study, all secondary causes could be excluded only in stable patients, as it is difficult to shift unstable patients for investigations such as a ventilation-perfusion scan, CT-scan, or CTPA. The initial diagnostic test is an echocardiogram when pulmonary hypertension is suspected based on history, physical examination, chest X-ray, or ECG to determine sPAP and to rule out underlying valvular cardiac etiology. The confirmation of diagnosis requires right heart catheterization and measurement of right atrial pressure, mPAP, and pulmonary capillary wedge pressure. Pulmonary vascular resistance (PVR) of more than three Wood units (240 dynes s/cm^5^) confirms the diagnosis [5]. In our patients, right heart catheterisation was not done, and diagnosis of pulmonary hypertension was based on echocardiography, as our hospital protocol does not include an invasive procedure requiring radiation exposure in pregnancy. LFTs and HIV were normal in our patients. Oxygen saturation varied from 68% to 99% in our patients. Chest X-ray findings varied from dilated pulmonary trunk, peripheral pruning, cardiomegaly, and left lower lobe collapse. Echocardiography revealed gross right ventricular dilatation, and CTPA was done to rule out thromboembolism. The risk of sudden death increases with pregnancy progression, as in our patient (case 6), who died within four to five weeks of diagnosis.

Maternal mortality associated with pregnancy is 30-50% [6]. In our study, maternal mortality was 36.36%. Most of the deaths occur in the third trimester, as seen in one of our patients who presented with intrauterine fetal demise and succumbed before definitive management could be undertaken. The highest risk is in the first 10 days postpartum, as also observed in our study (cases 1 and 9) [4,7]. This occurs due to changes in pulmonary vascular tone due to intravascular volume shifts, hypoxemia, elevated catecholamine levels, or sometimes thromboembolism. Thus, preconceptional counselling is of vital importance. Termination should be considered in cases of unplanned pregnancy or if the disease is diagnosed early in pregnancy. In our study, three patients presented in early pregnancy; two were already diagnosed to have PAH prior to pregnancy and were in stable condition, one of whom refused termination of pregnancy but had a good outcome; and one patient underwent MTP. One patient presented in the late second trimester in an unstable condition and refused termination of pregnancy. Her condition further deteriorated with the progression of gestation, and she died at 18 weeks of gestation. One patient presented in the third trimester with NYHA 4 status. Cesarean section was done in view of worsening cardiac condition, but she could not be salvaged.

Management of PAH encompasses the use of supportive measures and drug therapy after vasoreactivity testing. These include anticoagulation, oxygen supplementation for PO_2_ < 90%, and diuretic therapy. Endothelial cell activation may predispose to thrombosis, which may worsen right heart function. Anticoagulation should be given on a case-by-case assessment, keeping the medical as well as obstetric parameters of the patient in mind. On the one hand, the use of anticoagulation may help prevent thrombosis, but, on the other hand, management of anticoagulation in pregnancy may be challenging as it may result in hemorrhagic complications in the antepartum and peripartum periods. Acute vasoreactivity testing should be performed for these patients to determine the response to vasodilator therapy and to strategise treatment plans and prognostication. Responsiveness is defined as a decrease in mean PAP of 10 mmHg in response to a calcium channel blocker or a mean pulmonary artery pressure decline to 40 mmHg or lower. Inhaled nitric oxide, adenosine, and prostacyclin may also be used [5]. Calcium channel blockers are safe in pregnancy; however, sustained response to this therapy is seen in only about 10% of patients [8].

Targeted therapy for PAH involves the use of phosphodiesterase type 5 inhibitors (e.g., sildenafil, tadalafil), guanylate cyclase stimulants (e.g., riociguat), prostacyclins (e.g., epoprostenol, treprostinil, iloprost, selexipag), and endothelin receptor antagonists (e.g., bosentan, macitentan, ambrisentan). Though oral endothelin receptor antagonists have demonstrated the ability to improve pulmonary hemodynamics and exercise tolerance in the non-pregnant population by blocking vasoconstriction, their use in pregnancy is contraindicated due to teratogenic concerns demonstrated in animal studies [8].

Prostacyclins act as vasodilators, prevent aggregation of platelets, and reverse vascular remodelling, thus leading to improved functional class, exercise capacity, and survival rates. Nitric oxide and sildenafil have been used successfully during the intrapartum course [8]. In our study, all the patients were started on oxygen and anticoagulants, and some patients were already on sildenafil with good response, as shown in Table 1. Several case reports discuss the use of prostacyclins in pregnant patients. They require frequent administration by inhalation or continuous intravenous (IV) infusion in view of their short half-life. Rosalyn reported that epoprostenol therapy does not result in any fetal deformity or growth retardation during the last trimester of pregnancy [7]. Weiss et al. [9] and Albackr et al. [10] reported a successful treatment of a subject with inhaled iloprost intraoperatively and postpartum.

The ideal timing and route of delivery, as well as the mode of anesthesia, remain debatable. A planned elective delivery offers benefits in terms of availability of human and material resources and potential for delivery prior to cardiac decompensation development. A decrease in cardiac preload during the Valsalva manoeuvre and catecholamine release due to the physiologic stress of labor may precipitate cardiopulmonary collapse. During labour, epidural analgesia helps reduce pain and O_2_ consumption, controls vasodilatation for auto-transfusion, and hence improves hemodynamic consequences of labour [1]. Successful management during labor and delivery requires a multidisciplinary team approach with an obstetrician, pulmonologist, cardiologist, anesthetist, and experienced nursing staff. Bonnin et al. discussed 15 pregnancies affected by pulmonary hypertension, of which four cases were idiopathic (three underwent caesarean section and one had vaginal delivery) [11]. Three survived and one died three months postpartum. In our study, three (25%) pregnancies were delivered by vaginal route, five (41.66%) by caesarean section, one (8.33%) underwent MTP, and two (16.66%) died undelivered.

In a study by Bao et al. [12], there were eight cases of PAH with pregnancy, out of which four were idiopathic. All four patients died, reinforcing the fact that idiopathic PAH has faster progression and poorer prognosis in comparison to PAH due to congenital heart disease.

Successful maternal-fetal outcome following caesarean section in a patient with pulmonary arterial hypertension is related to the selection of anesthetic technique (epidural or general anesthesia); caesarean section was performed in patients who are unable to deliver by vaginal route or those who subsequently develop hemodynamic instability [6,13]. In our study, two patients (cases 2 and 10) had a caesarean section under general anesthesia.

Bedard et al. compared maternal mortality rates for 1997 to 2007 to 1978 to 1996 in pregnant patients with pulmonary hypertension and found a significant decrease in mortality rates(25 vs. 38%; p = 0.047) [14]. Data reported by Weiss et al. showed a maternal mortality rate of 17% in primary pulmonary hypertension. In a systematic review by Weiss et al. from 1978 to 1996, one maternal mortality rate was 36% in Eisenmenger syndrome, 30% in primary pulmonary hypertension, and 56% in women with secondary vascular pulmonary hypertension [15]. A study by Pieper et al. on the use of targeted treatments for pulmonary artery hypertension during pregnancy showed a substantial fall in mortality since an earlier review in 1998 (16% vs. 38%) and a further insignificant reduction in mortality since the recent review in 2009 (16% vs. 25%) [3]. In our study, patients who were already on treatment prior to pregnancy and those with lower PAP had better outcomes.

The neonatal survival rate is around 87-89%. Maternal hypoxemia results in growth retardation, preterm delivery, and stillbirth. In a series by Bonnin et al. [11], the neonatal survival rate was 85% (11 out of 13). Four neonates were under the 10th percentile. All children had normal development during the first year of follow-up. The neonatal prognosis is relatively good in contrast with the high maternal mortality rate [11]. In our study, out of two patients who presented in the postpartum period, one had a stillbirth, and one had a healthy baby. Out of seven patients who delivered in our institute, five (71.42%) had fetal growth retardation, one (14.28%) had intrauterine fetal demise, and four (57.14%) had preterm delivery.

Adverse neonatal outcomes have not been reported with the use of prostacyclins, as the majority of neonatal complications are attributed to prematurity resulting from maternal or fetal indications. A review of the literature by Xu et al. [6], Bédard et al. [14], and Weiss et al. [15] showed a neonatal survival rate of nearly 90%, with fetal growth restriction in 3-33% of pregnancies. Although there are no specific studies on the possible effects of epoprostenol on breastfeeding, prostacyclin is not orally active; hence, it does not seem to cause any effect if consumed in breast milk [8].

Demerouti et al. [16] reported two cases of PAH diagnosed during the third month after term pregnancies. One case was successfully treated with calcium channel blockers, and the other patient was treated with intravenous prostanoids with sildenafil, followed by atrial septostomy because of unresponsiveness to drugs, but had cardiac arrest. The first case presented in NYHA class II and the second in NYHA class IV, suggesting a possibility of better prognosis if PAH is detected early in the clinical course. Favourable functional class or mild pulmonary hypertension has a better prognosis [3].

Although the mortality rates remain high, recent evidence suggests that a multiprofessional approach and expert care may improve outcomes in patients who have been adequately counselled and still desire to continue pregnancy [17].

The present study discussed 12 patients with pregnancy with PAH and thus adds to the literature as this disease is a rare entity.

Limitations

A smaller number of patients and the retrospective nature of the study are major shortcomings of the present study. The small sample size may affect the generalizability of the study, but considering that the disease is a very rare entity, a prospective study of a longer duration may ensure a higher sample size increasing the validity of the study.

## Conclusions

Despite the most modern treatment efforts, the overall mortality in pregnant women with severe pulmonary hypertension is high. Therefore, pregnancy should still be discouraged in these patients. Detailed studies in pregnancy are limited in view of the non-invasive assessment of cardiac indices and the limited number of patients. Counselling should be done for these patients, explaining the high risk, and IUD and termination of pregnancy should be offered. However, some patients may wish to continue or plan a pregnancy despite being fully counselled regarding the high risk. Prostanoids should be started early in such patients. A multidisciplinary team approach involving an obstetrician with experience in managing high-risk pregnancy, an anaesthetist, a cardiologist, and a neonatologist is required for the best outcome. Despite optimal use of new treatment options, as well as a multi-professional approach, adverse outcomes for both mother and foetus are not uncommon. Advancements and research in treatment strategies are required to improve maternal and foetal outcomes, and contraception counseling is imperative.
